# Pedobarography: a novel approach to test the efficacy of treatments for lameness; an experience with mavacoxib in dogs with elbow osteoarthritis

**DOI:** 10.1186/s12917-019-1946-1

**Published:** 2019-06-11

**Authors:** Sergio López, José M. Vilar, Mónica Rubio, Joaquín J. Sopena, Angelo Santana, Oliver Rodríguez, José A. Rodríguez-Altonaga, José M. Carrillo

**Affiliations:** 1 Departamento de Patología Animal, Universidad de las Palmas de Gran Canaria, Arucas, Las Palmas, Spain; 2 Instituto Universitario de Investigaciones Biomédicas y Sanitarias, Universidad de las Palmas de Gran Canaria, Arucas, Las Palmas, Spain; 3 Departamento Medicina y Cirugía Animal, Cátedra García Cugat, Universidad CEU Cardenal Herrera, Valencia, Spain; 4 Departamento de Matemáticas, Universidad de las Palmas de Gran Canaria, Las Palmas, Spain; 5 Departamento de Medicina, Cirugía y Anatomía Veterinaria, Universidad de León, León, Spain

**Keywords:** Balance, Pedobarography, Dog, Mean pressure, Peak pressure, Paw, Lameness, COP (center of pressure)

## Abstract

**Background:**

Pedobarographic analyses detect pressure redistribution among limbs and within limbs in humans, equids and dogs. The main objective of this study was to assess the usefulness of a set of pedobarographic parameters for the detection of lameness, as well as for its suitability for assessing the effects of therapies against osteoarthritis in dogs. With this purpose, eleven large-breed lame dogs with unilateral osteoarthritis due to elbow dysplasia were evaluated using a pressure platform prior to (D0) and after 3 months (D90) of treatment with mavacoxib, a COX-2 selective NSAID. The obtained parameters were: pressure distribution between lame and sound limbs, as well as paw area, mean pressure, and peak pressure of both lame and sound limbs.

**Results:**

The results showed statistical differences in all these parameters between lame and sound limbs at D0; however, at D90, differences were significantly decreased as result of the treatment, indicating a substantial functional recovery under the study design conditions.

**Conclusions:**

The provided data prove the suitability of this novel technique in canine models for the quantitative and objective assessment of lameness, but also for the evaluation of treatments for lameness caused by articular pain.

**Electronic supplementary material:**

The online version of this article (10.1186/s12917-019-1946-1) contains supplementary material, which is available to authorized users.

## Background

Elbow dysplasia (ED) is one of the most frequent causes of lameness of articular origin in a dog’s forelimbs, which often evolves to osteoarthritis (OA). This deteriorating disease results from developmental conditions [1], and could affect up to 20% of dogs beyond one year of age [2].

Regarding pain measurement, different protocols have been published not only for its detection, but also for assessing the efficacy of different treatment options against OA. With this aim, veterinary clinicians test pain degree using a scoring panel with parameters as vocalization, level of activity, functional deficit, joint mobility, and/or reaction to manipulation [3]; however, the subjectivity due to intrinsic and extrinsic factors does not allow a fully objective evaluation [4].

Biomechanical evaluations of gait, especially by kinetic methods as force platforms, have been shown as an objective tool for the detection of locomotor alterations due to pain. Essentially, the detection of lameness and its evolution when it has been surgically or medically treated has been reported [5, 6]. This methodology has also proven how poor subjective scoring systems are in assessing lameness in dogs [6–9].

More recently, pressure platforms/walkways have shown potential in lameness diagnoses by providing additional parameters, contributing to a more integrative vision of gait deficits [10]. This is because pain causes postural modifications [11]; the consequence of this modification is an asymmetric center of pressure (COP) sway and a resultant redistribution of pressure when limbs are supported on the floor.

The study and graphical representation of pressure imbalances is defined as pedobarography; these data can be obtained at stance (static pedobarography) or with a moving subject (dynamic pedobarography) [12]. The usefulness of this technique has been widely proven in human medicine, rehabilitation, and/or sport fields [12–14].

In domestic animals, pressure-dependent data have been published in the last few years, providing valuable data as mean and/or peak pressure in paws during weight bearing [15] and paw pressure contact area [10]; this is based in the principle that dog pads or equine hoofs spread as weight bearing increases [16–18]. More recently, and together with additional parameters as statokinesiograms, this technique has been used to assess the effectiveness of a PRP-derivate in OA dogs [10].

Therapy against OA should focus on reducing joint pain, delaying the disease progression, and restoring joint function at a maximum with an overall improvement in the quality of life [19]. With this in mind, mavacoxib (Trocoxil, Zoetis, Spain) is an orally administered COX-2 inhibitor, which has a mean half-life of 44 days in dogs [20]. This long half-life enables a dosage of 2 mg/kg once monthly after two initial doses 14 days apart [3, 21]. In addition, its effectiveness in vitro against cancer has been proven [22]. Its efficacy in OA dogs has been previously tested [1], even with objective data obtained with force platforms [3, 23].

For all the reasons above, the aim of this paper is to objectively assess the suitability of dynamic pedobarography in the detection and quantification of lameness, as well as to assess the efficacy of mavacoxib in the functional improvement in dogs with unilateral ED by means of DP.

## Results

From the 17 initially included client-owned lame dogs with confirmed unilateral ED, 3 were discarded due to the inability to remain immobile during postural exam; another dog was unable to walk on the leash properly, and one more due to an alteration in a blood biochemical profile. A total of 11 dogs (four Labrador, one Rottweiler, three Presa Canario, and three mixed dogs) were finally included in the study. Individual characteristics are summarized in Table 1.

**Table 1:** Dogs’ individual basic profiles

Dog #	Breed	Age (years)	Weight (Kg)	Gender
1	Labrador	6	33	M
2	Rottweiler	5	51	M
3	mixed	9	33	F
4	mixed	3	30	M
5	mixed	4	31	F
6	Labrador	6	30	M
7	Labrador	8	31	M
8	Labrador	4	30	M
9	Presa Canario	4	41	F
10	Presa Canario	7	49	M
11	Presa Canario	3	42	F

*M* Male, *F* Female

**Fig. 1:**
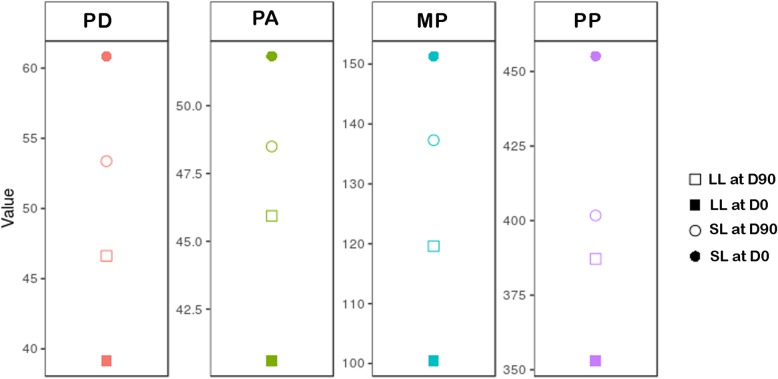
Comparison of differences between LL and SL values at D0 and D90 for PD, PA, MP, and PP. Solid circle and quadrate represent mean values of SL and LL, respectively, at D0. Empty circle and quadrate represent mean values of SL and LL, respectively, at D90. Differences decreased at D90 for all four parameters. Value units: PD (%); PA (cm^2^); MP and PP (kPa)

The animals had a mean body weight of 36.45 ± 7.92 Kg and a mean age of 5.36 ± 2.01 years. The mean values ± SD and 95% confidence intervals of all obtained parameters are summarized in Table 2. Data were all normal (*p* ≥ 0.1) and homoscedastic (*p* ≥ 0.11).PD, PA, MP, and PP values in both LL and SL showed differences between D0 and D90 (*p* ≤ 0.0001 in all cases), which means an increase in LL values and a consequent decreasing in SL values. Differences between SL and LL decreased from D0 to D90 (p ≤ 0.0001 in all cases); this could be interpreted as an improvement in LL function (Fig. 1). However, these differences remained for PD, PA, and MP (p ≤ 0.0001 in all cases), which means that full recovery was not reached. In change, PP values at D90 showed no differences (*p* = 0.1487), although the high variability (high SD) in this parameter could explain this fact.

**Table 2:** Pedobarographic Parameters in Dogs, Expressed as Mean ± SD, and 95% Confidence Intervals

Day	LL	SL	% Difference
PD (%)			
D0	39.15 ± 2.47	60.85 ± 2.47	21.71^+^ ± 4.94%
	(37.91, 40.37)	(59.23, 62.48)	(19.25, 24.16)
D90	46.62 ± 2.16	53.38 ± 2.16	6.75^+^ ± 4.33%
	(45.39, 47.85)	(52.01, 54.74)	(4.29, 9.20)
PA (cm^2^)			
D0	40.59 ± 2.32	51.83 ± 1.66	23.56 ± 6.38%
	(39.95, 41.74)	(51.11, 52.32)	(21.05, 26.07)
D90	45.94 ± 2.09	48.53 ± 1.33	5.81 ± 6.07%
	(44.92, 46.71)	(47.92, 49.13)	(3.30, 8.32)
MP			
D0	100.44 ± 5.28	151.33 ± 10.69	40.24 ± 9.21%
	(98.08, 102.79)	(147.59, 155.07)	(36.48, 44.00)
D90	119.59 ± 5.51	137.30 ± 5.12	13.80 ± 6.92%
	(117.29, 121.94)	(133.56, 141.04)	(10.265, 17.56)
PP			
D0	352.97 ± 19.66	455.15 ± 35.79	53.81 ± 30.75%
	(343.13, 362.81)	(442.32, 467.03)	(39.25, 68.36)
D90	387.17 ± 28.39	401.73 ± 25.17	11.39 ± 30.34%
	(374.32, 399.01)	(385.86, 417.81)	(−3.09, 25.86)

*D0* Before treatment, *D90* Three months after the first oral application of treatment, *LL* Lame limb, *SL* Sound limb, ^+^ In regards to the ideal symmetry (i.e., 50% for each limb)

**Fig. 2:**
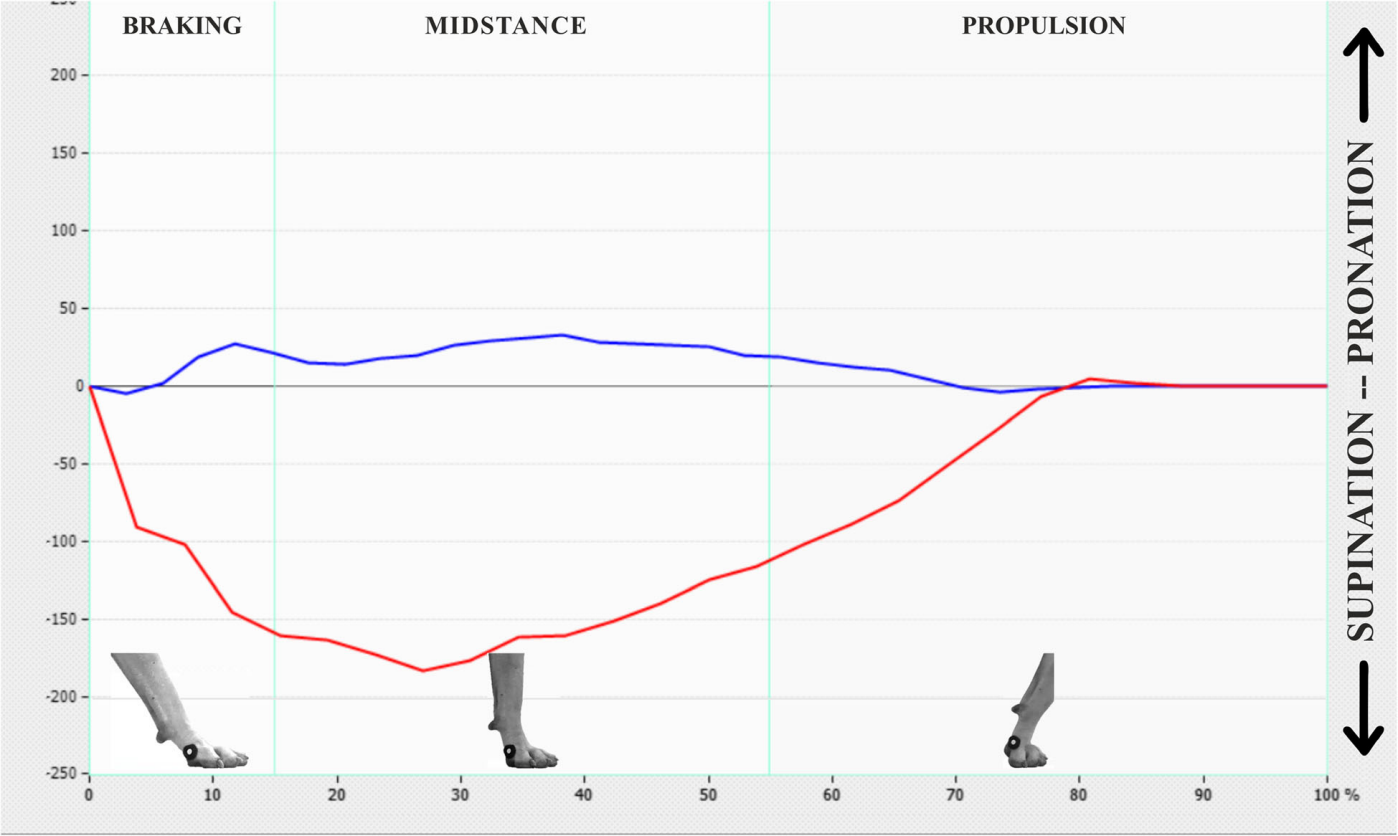
Graphic shows lateromedial displacement of a sound (blue) and a lame (red) limb during the support phase at D0. Horizontal axis is expressed in percentage in terms of time of the whole support phase. Vertical axis is represents in kPa the lateromedial deviation. Supination in the LL is evident

**Fig. 3:**
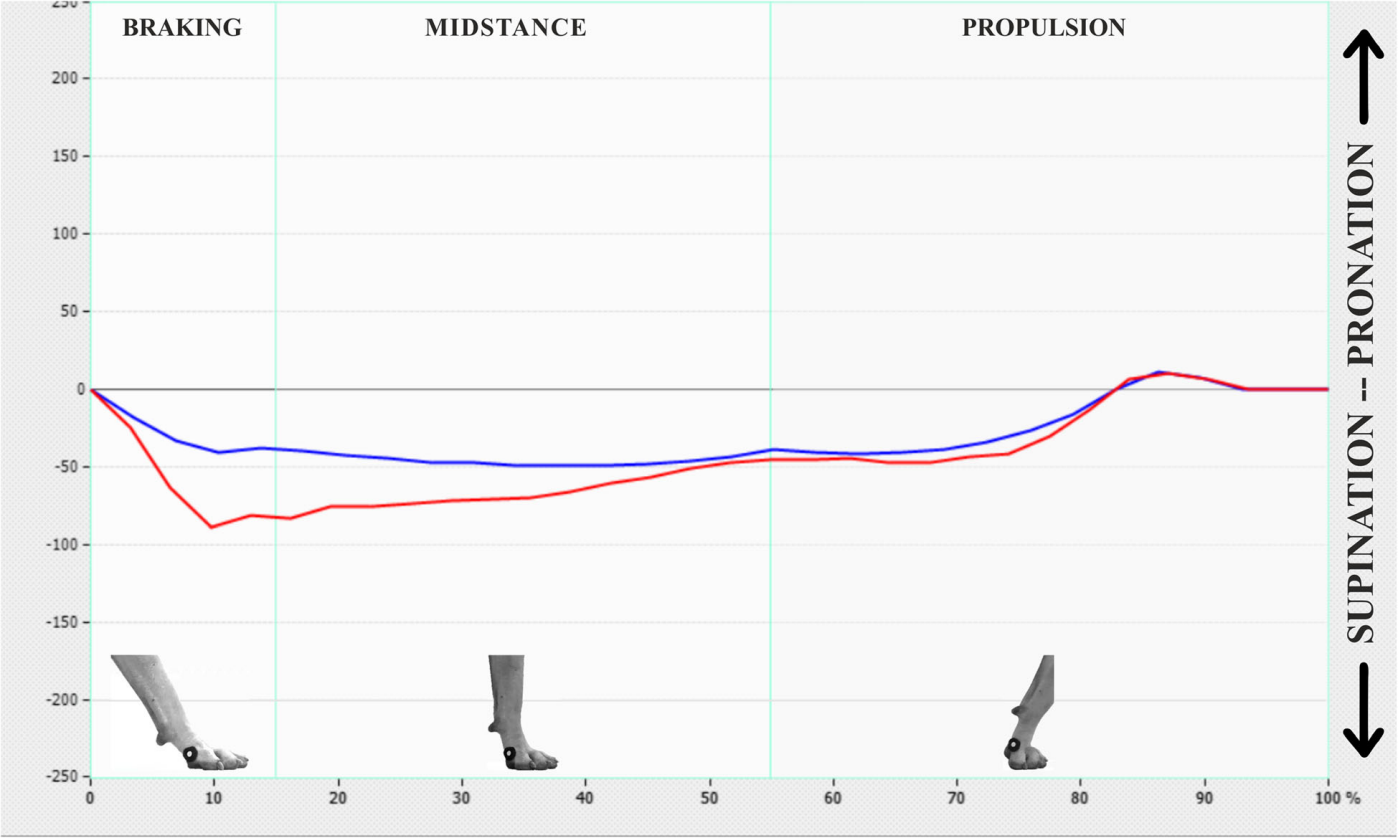
Graphic shows lateromedial displacement of a sound (blue) and a lame (red) limb during the support phase at D90. Horizontal axis is expressed in percentage in terms of time of the whole support phase. Vertical axis is represents in kPa the lateromedial deviation. Patterns in both SL and LL are similar

Graphical comparison of lateromedial balance between LLs and SLs allowed us to see a marked instability during the support phase in LLs (Fig. 2); after 3 months of treatment, limb stability increased, becoming similar to SLs (Fig. 3).

In addition, videosequences during the support phase at walk demonstrate important differences in pressure distribution. At D0, in SLs the COP path is symmetric; however, in LLs, the COP path shows a lateral migration in an attempt to alleviate pain during support (Additional file 1). This lateral COP path migration in the paws is less evident at D90 (Additional file 2).


**Additional file 1:** Video sequence of a whole support phase of a LL (left) and a SL (right) at D0. COP (black and red point) displaces more laterally in LL. This is more evident when COP paths (rose line) from both LLs and SLs are compared. In sound limbs cross between the third and fourth digital pads, while in LL is over the fourth digital pad. (MP4 1067 kb)



**Additional file 2:** Video sequence of a whole support phase of a LL (left) and a SL (right) of the same dog at D90. COP path in LL runs now more symmetrically, between the third and fourth digital pads. (MP4 1260 kb)


## Discussion

In the present study, the efficacy of a set of new, objective pedobarographic parameters derived from the imbalances at walk to detect unilateral lameness in dogs has been proven. Under the authors’ knowledge, this should be the first time that these parameters are used for lameness detection, at least in dogs.

Considered the “gold standard” in objective gait assessment, force platforms have being classically used to obtain the peak vertical force and vertical impulse as parameters for lameness detection. However, in a recent report, this technique was compared with subjective evaluation and inertial sensors in horses with subtle and mild lameness; the authors conclude that, ideally, lameness detection should be performed from different approaches [24]. In line with this, pedobarography provides a set of different parameters capable to assess lameness in a more integrative way from different points of view, keeping up the coherence with force platform findings [25].

Another important advantage of these technologies is that they are uninfluenced by subjective factors (observer, questionnaires, etc.) [4, 26, 27] when the efficacy of treatment in OA dogs is being assessed [28].

As consequence, the obtained results proved that significant differences can be found in all studied parameters when LLs and SLs are compared, and, additionally, that this difference decreases when an effective treatment is settled. However, in the case of PP values, its high variability probably did not detect differences between limbs at D90; therefore, the interpretation of PP results should be taken cautiously. Our findings slightly differ when compared with another previous work, where dogs with hip dysplasia belonging to a unique breed were treated with mavacoxib. In this case, functional recovery seemed to be complete [23]. Different breed selection, different affected joints, and study design could explain this fact.

The assessment of pressure modifications due to posture imbalances in lame dogs before and after treatment has been previously reported, although, in this case, the treatment consisted of a platelet-rich plasma derivate in elbow-diseased dogs and included only postural, static data [10]. If both therapy outcomes are compared, it could be said that mavacoxib efficacy is higher at the same checking period (D90) with animals with similar conformation. The same occurs when compared with other NSAIDs [3].

Regarding the administration regimen, its monthly dosage makes its administration easier, which should increase owner compliance in treating their dogs [22]. On the other hand, previous safety studies confirmed that the dosage regimen used in this study provides the highest long-term efficacy against OA with a minimum of adverse effects [21]; in this sense, in a recent study with 62 treated dogs, the most frequently cited side effect was related to digestive tract disorders, all of them categorized as minor and transient [29].

In spite of the excellent results shown, this experimental design had limitations. First, results were obtained by recording the dogs after several hours of resting, which could improve the lameness. Although the time of examination was relatively short, some dogs showed an evident lameness after some minutes when owners allowed free play after the platform test. In this sense, to perform the posturographic test on these animals after a standardized period of exercise should be a major requirement.

Second, the animals’ behavior was an important limiting factor during the data collection because dogs with nervous temperaments refused to walk on the platform, requiring more time to familiarize themselves with the platform or, if unsuccessful, removal from the study.

Similar problems were referred to in another report, while performing the statokinesiograms, where dogs had to remain completely immobile over the platform during the 10 s recorded lapse [10]. In this case, we chose 20s to record PD from a wider range of time.

Third, with these “standard resolution” pressure platforms, the comparison of posturographical parameters provided here between lame and sound dogs could only be obtained from animals of approximately 20–25 kg or more; with dogs of smaller size or cats, significant differences between obtained data could be hard to find. For animals weighing less than 20-25 kg, high-resolution platforms are necessary to increase the sensitibity to pressure changes [30].

Fourth, and last, although lateromedial imbalance and COP sway in LLs could not be quantified, the graphic representation of these events provides a suitable proof of joint instability and body posture modification, as well as its improvement after mavacoxib treatment. The observed increased supination in LLs has been previously reported in dogs with the same disease using kinematic methods [31]. A possible reason could be that articular pain leads to a lack of muscular mass and/or strength due to limb misuse, lacking joint stability during support.

## Conclusion

Pedobarography has shown to be suitable and reliable for the assessment of unilateral lameness in dogs with ED. The functional recovery when a treatment was established could also be assessed.

## Methods

### Animals

The inclusion criteria were adult dogs with weight ≥ 30 Kg, age ≥ 3 years, and presence of lameness due to unilateral ED. The animals should be free of any concurrent systemic or orthopedic disease, and with a treatment-free interval of a month.

Accordingly to criteria, three standard radiographs views [32] were taken under sedation with dexmedetomidine 10 ± 20 μg/kg (Dexdomitor, Zoetis, Spain) of both elbows of each animal in order to confirm that animals were unilaterally affected by ED. Radiographic signs should be consistent with subtrochlear sclerosis, unclear delineation of the medial coronoid process, and/or presence of osteophytosis. The same researcher (JAR) performed the radiological analysis.

Additionally, all dogs also underwent a complete clinical examination of the locomotor system including fore and hindlimbs and hematologic in order to create blood and urine biochemical profiles to assure that general health was in normal ranges and ED was the only reason for gait modifications.

Once the animals of the study group met the inclusion criteria, each was treated with oral mavacoxib at a dosage of 2 mg/Kg once monthly, after two initial doses 2 weeks apart. All the dog owners continued treating their dogs with mavacoxib after experimental phase was concluded.

### Pedobarographic analysis

A pressure platform (EPS-R®, Loran Engineering, Bologna, Italy) was placed dissembled within a 7 m rubber runway carpet. The device contains a total of 2096 pressure sensors of 1 cm^2^ distributed in an area of 48 × 48 cm. The range of pressure was 30–400 kPa and had a sampling frequency of 100 Hz.

The pressure platform was interfaced with a dedicated computer using Biomech® (Loran Engineering, Bologna, Italy). This software allowed the recording, numerical and graphic conversion, as well as the storage of data. Biomech® also allowed the discarding of data post-acquisition from those sensors, which recorded data from different limbs within the same gait cycle.

The obtained parameters were: pressure distribution between limbs (PD, %), paw area (PA, cm^2^), mean pressure (MP, kPa) and peak pressure (PP, kPa), lateromedial limb displacement (LMD, kPa), and Pedobarographic patterns during the support phase, although these last two events could not be statistically treated due to intrinsic reasons of the parameters or software limitations to obtain the numerical data. Data acquisition was performed before treatment (D0) and at day 90 (D90) after treatment started.

For PD, dogs were placed in a square standing stance (with their limbs in a rectangular position and the head held directly in front), while the dog’s owner remained in front of the animal to attract the dog’s attention at a close distance. When the dogs seemed relaxed, data collection began and continued for 20 s.

PA, MP, PP, LMD and pedobarographic patterns were obtained walking the dogs on a leash guided by their owners over the pressure platform at walk. The animals were previously given time to get familiar with walking on the device, generally taking 5–10 min.

Three valid trials were obtained from each dog. A trial was considered valid if velocity of the dogs was within a range of 1.6 ± 0.3 m/s and acceleration ≤ ± 0.2 m/s2.These parameters were measured by a motion sensor (Pasco, California, USA) in such a way that measurements out of these limits were discarded. Other validity parameters were that the limb should fully contact the pressure platform and the dog should walk close to its owner without pulling on the leash.

### Statistical analysis

A linear mixed effects model was used for the analysis of data, where the treatment (D0/D90) and the status of the limb (lame (LL)/sound (SL)) were considered as fixed factors and the dog as a random factor. The model is of the form:

$$ {y}_{itlk}=\mu +\beta {I}_t+\gamma {I}_l+\delta {I}_{t\cdotp l}+{b}_i+{\varepsilon}_{itlk} $$

where *y*_*itlk*_ is the value of the *k*-th measurement (*k* =1,2,3) of the variable (parameter) *y* in the limb *l* (LL/SL, being *I*_*l*_ = 1 if lame and *I*_*l*_ = 0 if sound) of the *i*-th dog taken at time *t* (D0/D90) *I*_*t*_ = 0 at D0 and *I*_*t*_ = 1 at D90). Parameters of the model are interpreted the following way:

- *μ*: mean value of the variable in the SL before treatment.
- *β*: effect of the treatment.
- *γ*: difference LL/SL.
- *δ*: interaction between limb and treatment (increase/decrease)
- *b*_*i*_: random effect of dog *i*. We assume that *b*_*i*_ ≈ *N*(0, *σ*_*b*_)

Ninety-five percent confidence intervals (95% CI) were calculated for the model parameters and the differences between limbs. Normality in the residuals was checked using the Shapiro-Wilk test. Homoscedasticity of the residuals was checked by the Levene’s test. For all tests, a significance level of 5% was used.

For comparison between LL and SL in PA, MP, and PP, the difference was calculated using the following formula:

$$ \varDelta \%=200\ast \left( SL- LL\right)/\left( SL+ LL\right) $$

where the difference represents a symmetry index; 0% should represent perfect symmetry [33].

Differences with *P*-values < 0.05 were considered statistically significant.

Statistical analysis was performed with ‘R’ statistical language and environment, version 3.3.2. (https://www.R-project.org/).

## Data Availability

All data supporting our findings are included in the manuscript. If readers need additional information and/or data sets, they will be provided by the corresponding author upon reasonable request.

## Abbreviations

COP: Center of pressure
ED: Elbow dysplasia
LL: Lame limb
LMD: Lateromedial displacement
MP: Mean pressure
OA: Osteoarthritis
PA: Paw area
PD: Pressure distribution
PP: Peak pressure
SL: Sound limb
